# Investigating the Potential Double-Edged Score of Immigration-Related Stress, Discrimination, and Mental Health Access

**DOI:** 10.3390/ijerph21030373

**Published:** 2024-03-20

**Authors:** Arthur R. Andrews, Kevin Escobar, Sandra Mariely Estrada Gonzalez, Sara Reyes, Laura M. Acosta

**Affiliations:** 1 Department of Psychology, University of Nebraska-Lincoln, Lincoln, NE 68588, USA; kescobar2@huskers.unl.edu (K.E.); laura.acosta@huskers.unl.edu (L.M.A.); 2 Institute for Ethnic Studies, University of Nebraska-Lincoln, Lincoln, NE 68588, USA; 3 Department of Psychological Science, University of Texas, Rio Grande Valley, Edinburg, TX 78539, USA

**Keywords:** discrimination, immigration stress, depression, PTSD, care access

## Abstract

Prior work has suggested that discrimination and immigration-related stress may impede mental health care seeking and utilization among Latinx populations. These effects may be more nuanced as both discrimination and immigration-related stress may increase symptomology, particularly post-traumatic stress disorder (PTSD) and depression. Both symptoms may, in turn, prompt attempts to seek care. The current study examined the direct effects of discrimination and immigration-related stress on care access, as well as potentially indirect effects with PTSD and depression symptoms as mediators. Interviews and online surveys were completed with 234 Latinx residents of the Midwest, assessing everyday discrimination, discrimination in healthcare, PTSD symptoms, depression symptoms, current mental health utilization, and previous unsuccessful attempts to seek care. Direct effects of discrimination and immigration-related stress were largely unrelated to care access variables. Discrimination in healthcare settings predicted both unsuccessful attempts to seek care and current use, but this effect was positive. The indirect effect was largely supported, but only for PTSD symptoms and not depression. Results indicate that further investigation is necessary to understand the direct effects of discrimination on care access. Further, discrimination and immigration-related stress may indirectly prompt attempts to seek mental health care.

## 1. Investigating the Potential Double-Edged Score of Immigration-Related Stress, Discrimination, and Mental Health Access

Discrimination and immigration-related stress appear to increase mental health symptomology across multiple domains, including depression and PTSD symptoms [1,2,3,4]. Despite the increase in symptomology and the potential need for services, both discrimination and immigration-related stress may increase the avoidance of healthcare systems for Latinx populations, especially immigrants and those close to them [5,6]. Based on healthcare utilization models, like the Health Belief Model [7,8], this may place many Latinx populations in a double bind wherein discrimination and immigration-related stress prompt greater need for care but also impede access to that care. The current study will investigate these compounding relationships based on the Health Belief Model in a predominantly immigrant sample of Latinx residents in the Midwest. It also extends prior work as most research examining the effects of discrimination on mental health symptoms, PTSD in particular, focus on African American/Black populations [9,10].

## 2. The Health Belief Model and the Potential Roles of Discrimination and Immigration Stress 

The Health Belief Model is a theoretical model outlining the factors that prompt health behavior change [7]. Much of the Health Belief Model research has focused specifically on healthcare utilization [11,12]. The model overall outlines six factors as necessary for healthcare utilization: (1) perceived susceptibility, (2) perceived severity, (3) perceived benefits, (4) perceived barriers, (5) cue to action, and (6) self-efficacy [7]. According to this model, only perceived barriers would impede attempts to care, while each of the other five would increase care-seeking behaviors. In a mental health utilization context, perceived benefits are defined as including the perceived benefits of seeking treatment and of treatment itself [11]. Said differently, this is the perception that available/accessible treatment will be effective in addressing symptoms. Relatedly, self-efficacy, in this case, would include the perceived ability to access treatment and perform the tasks involved during treatment. Multiple investigations have supported the Health Belief Model in predicting mental health service utilization [13,14,15]. In particular, these studies often find that perceived benefits to treatment are the strongest predictor of seeking mental health services and that perceived barriers may work in conjunction with perceived benefits (e.g., perceived barriers moderate the effect of perceived benefits) [13,14,15]. 

As outlined in more detail elsewhere [16], discrimination may diminish the perceived effectiveness of services and increase perceived barriers to care by increasing the social cost of care. This applies to both discrimination in general, given the lack of representation in healthcare, and discrimination in healthcare settings specifically. Immigration-related stress may operate in a similar fashion in that many factors involved in immigration-related stress may increase multiple perceived barriers, including financial and social costs [5]. From the prior literature, these costs may include fear of deportation and fear of unfair treatment in healthcare settings [5,17]. Both fear of deportation and fear of unfair treatment in healthcare due to immigration status may prompt avoidance of mental health utilization [17]. Although much of this literature has focused on undocumented immigrants, such fear may extend to other Latinx immigrants and those close to them (e.g., fear of deportation of a loved one) [18,19,20]. Further, due to assumptions surrounding immigration status, many Latinxs born in the U.S. may also experience several immigration-related stressors [18]. Further, the effects of immigration-related stress have less often been examined quantitatively and not within the context of the Health Belief Model. 

In contrast to immigration-related stress and discrimination, symptom recognition for both PTSD and depression is, by definition, a necessary component of perceived severity, which would be posited to increase utilization. Thus, self-reporting of symptoms is a direct indicator of perceived severity. Taken together with the extant literature on the effects of discrimination on depression and PTSD [3,9,21,22,23], this suggests that these symptoms may, therefore, operate as potential mediators of a positive indirect effect of discrimination on seeking mental health care. To date, the potential indirect effect is not well-established. Thus, testing this indirect effect among Latinx populations would represent a novel addition to the research literature and potentially add nuance regarding the conflicting roles of discrimination on care access. 

Though examined less frequently, immigration-related stress may operate similarly. Prior work has indicated that immigration-related stress is associated with higher depression symptoms [1,4]. Immigration-related stress may also exacerbate PTSD symptoms, though this has been examined less frequently. Theoretically, immigration-related stress may exacerbate symptoms such as hypervigilance, avoidance, and hyperarousal for Latinx immigrants and those around them. This may result from persistent fears of deportation or challenges fulfilling essential life functions because of immigration status (e.g., transportation because of fear of police interactions).

## 3. Purpose

The current study sought to quantitatively examine the potential dual roles of discrimination and immigration-related on mental health care seeking. This represents a novel step in examining the potential indirect effects of discrimination and immigration-related stress on mental health care seeking. This is an important step, given prior work indicating that discrimination and immigration-related stress may impede care seeking. Thus, taken together, both discrimination and immigration-related stress may produce conflicting direct and indirect effects on care seeking. To test these hypotheses, the study first examined the direct effects of both factors in potentially reducing care seeking and, subsequently, care utilization. Second, it examined the indirect effects of both factors, with PTSD and depression symptoms as potential mediators. It was hypothesized that both discrimination and immigration-related stress would have negative direct effects on mental health care seeking and care utilization. It was further hypothesized that both discrimination and immigration-related stress would evidence positive indirect effects on care seeking and care utilization with PTSD and depression symptoms as mediators. Specifically, discrimination and immigration-related stress would have positive direct effects on depression and PTSD symptoms. Depression and PTSD symptoms would, in turn, have positive direct effects on care seeking and care utilization.

## 4. Materials and Methods

### 4.1. Participants

Participants were 234 Latinx residents of the Midwest from cities and towns of population sizes ranging from approximately 5000 to approximately 300,000. The majority were born outside the U.S. (*n* = 197; 87.2%) and were cisgender women (*n* = 165; 70.2%). The largest proportion of participants were born in Mexico (*n* = 91; 38.7%), followed by Cuba (*n* = 37; 15.7%), Guatemala (*n* = 25; 10.1%), Honduras (*n* = 14; 5.9%), and Colombia (*n* = 10; 4.2%). Fewer than 10 participants were born in El Salvador, Ecuador, Peru, Venezuela, Nicaragua, and Argentina. The average age was 42.52 years. The majority of participants indicated they completed a high school degree or more (*n* = 178; 78.1%). Additional background information can be found in Table 1.

### 4.2. Procedure

Participants were recruited separately through phone and internet surveys. For the phone surveys, participants were recruited through multiple local community agencies that served Latinx populations. Participants were also recruited via respondent-driven sampling, in which existing participants were offered incentives for recruiting additional participants (USD 10 gift card for each participant recruited). Prior participants were not informed as to who completed the survey to preserve the confidentiality of participation. Recruitment for the phone survey occurred from November 2020 to August 2021. Recruitment for the internet survey occurred through a university extension agency, which provides education and resources to the local community. Recruitment for the internet wave occurred from January to April 2022. In both cases, surveys were completed as a larger study focusing on COVID-19, stress exposure, mental health, and healthcare access. In both internet and phone surveys, participants were able to complete the study in either English or Spanish. These procedures were approved by the Institutional Review Board (IRB) at the University of Nebraska-Lincoln

### 4.3. Measures

#### 4.3.1. Discrimination

Discrimination was measured using two questionnaires. The six-item Everyday Discrimination Scale (EDS) [24] was used to measure general experiences of discrimination in multiple contexts, and the seven-item Discrimination in Medical Settings Scale (DMS) [25] was derived from the EDS such that it represents a context-specific version of the EDS [25]. Both scales have demonstrated good internal consistency and concurrent validity [24,25,26,27]. Both have also been used to measure experiences of discrimination in Latinx samples [24]. The EDS demonstrated good internal consistency in the current sample (Cronbach’s α = 0.96), as did the DMS (Cronbach’s α = 0.94).

#### 4.3.2. Immigration-Related Stress

Immigration-related stress was measured using the five-item Lack of Legal Immigrant Status subscale from the Stress of Immigration Survey [28]. Only this subscale was used due to brevity. Items assess common fears related to precarious immigration status for the participant, with multiple items also referencing difficulties for family members. Items ask participants “how much stress or worry (they) have experienced” as a result of the fear that they or a family may be deported, not having the correct documents when accessing services, not having a license to drive, difficulties accessing healthcare for the participant or family because of documentation, and difficulties returning to visit their home country. As such, many of these items apply to undocumented and documented immigrants and their families. The scale evidenced strong internal consistency in the current sample (Cronbach’s α = 0.95).

#### 4.3.3. Post-Traumatic Stress Disorder (PTSD)

PTSD was measured using an abbreviated version of the PTSD Checklist for DSM-5 [29]. The PCL-5 assesses symptoms of PTSD taken directly from the DSM-5 diagnosis and asks participants to rate their frequency from “not at all” (0) to “extremely” (4). The PCL-5 has demonstrated good internal consistency [29]. A Spanish language version has also evidenced good internal consistency with Mexican and Central American samples [30,31]. An abbreviated version was used to accommodate requests for brief surveys from recruitment partners and to minimize the study burden. Five items were initially included (Item B1-Intrusive thoughts, Item B4-Emotional Cue Reactivity, Item C1-Avoidance of external reminders, Item E1-Irritability or anger, and Item E4-Easily startled). However, confirmatory factor analyses indicated that Item E4 had only a moderate loading onto a single item factor (λ = 0.49), and removing it yielded a good model fit across most indicators, χ^2^ (2) = 0.32, CFI = 1.00, RMSEA < 0.001 (90% CI = <0.01–0.07), and SRMR = 0.01. Thus, only four items (B1, B4, C1, and E1) were included in the present analyses. 

#### 4.3.4. Depression

Depression symptoms were assessed using a modified version of the nine-item patient health questionnaire (PHQ-9). The PHQ-9 is among the most widely used measures of depression, including in several large international studies of depression [32,33,34,35]. The PHQ-9 typically contains nine items, but the final item regarding suicidality was removed so that the study could be more readily administered in an online context. This modification has been used in prior studies where the measure still demonstrates strong internal consistency and concurrent reliability [36]. The Spanish translation of the PHQ-9 has also demonstrated good internal consistency and concurrent validity [34,35]. The eight-item version demonstrated good internal consistency in this sample (Cronbach’s α = 0.91).

#### 4.3.5. Mental Health Access

Mental health access was measured using two items. The first asked participants if they had sought services since the COVID-19 pandemic began but were unable to receive them. The second asked if participants were currently receiving services. These two items, therefore, represent unsuccessful attempts to receive care and current mental health use, respectively. Participants answered either “yes” or “no”, which were then dichotomously coded as “1” and “0”, respectively. As part of exploratory follow-up questions, participants who indicated they had been unable to receive services were asked to identify barriers that prevented them from receiving care. 

#### 4.3.6. Demographics

Participants also completed items regarding gender, age, U.S. nativity/country of origin, and education.

### 4.4. Data Analyses

**Figure 1:**
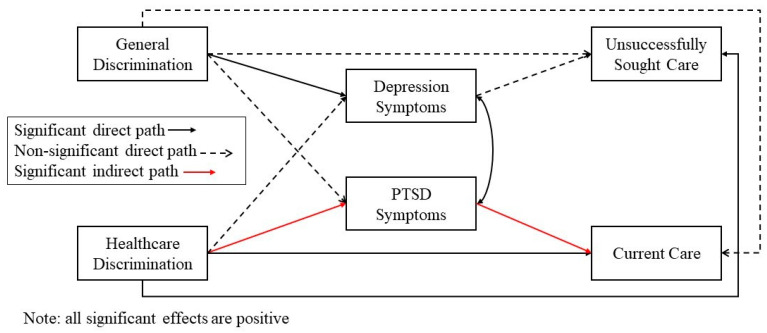
Path model of direct and indirect effects of discrimination on mental health access.

**Figure 2:**
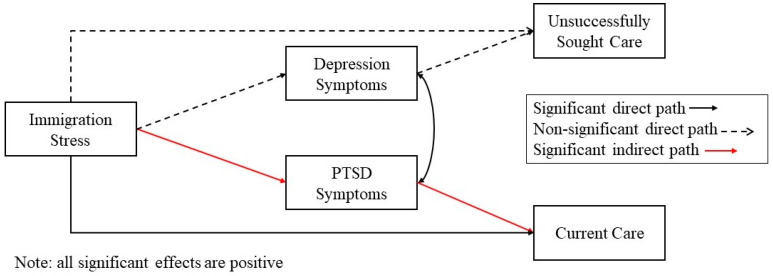
Path model of direct and indirect effects of immigration stress on mental health access.

Study hypotheses were tested using two separate path models in which depression and PTSD symptomology were examined as mediators between experiences of discrimination (model 1)/immigration stress (model 2) and the two mental health utilization variables. These models are depicted in Figure 1 and Figure 2, respectively. To test specific hypotheses, the direct effects of discrimination/immigration stress variables on both PTSD and depression were examined, as were the direct effects of PTSD and depression on both mental healthcare access variables and the constituent indirect effects. Additionally, the direct effects of discrimination variables on mental healthcare access variables were also tested. Age, gender, and education were included as covariates in the model. Significance for all effects, including indirect effects, was examined using bootstrapped confidence intervals with 500 sample draws. Because the model includes two dichotomous variables as outcomes and a mediational structure, the Monte Carlo integration was used, and frequently used model fit indices (e.g., CFI) are not available. However, the mediational models were compared against simpler models using log likelihood ratio tests. Specifically, it was compared against two models with no mediational paths in which symptomology was either only outcomes (with only discrimination as predictors) or only predictors (with only healthcare access variables as outcomes. The mediational model was not significantly different from either of the two simpler models (*p*-values > 0.05). Thus, given the specific hypotheses regarding the indirect role of symptomology, the mediational model was retained and included here. 

Prior to completing analyses, analytic assumptions and missing data were assessed. Only items from the EDS evidenced significant missingness (>10% missing). All missing data were estimated using full information maximum likelihood estimation, which has been associated with reduced bias relative to other methods [37]. All other analytic assumptions were met.

## 5. Results

### 5.1. Discrimination as a Predictor of PTSD and Depression

In the discrimination model, discrimination in healthcare settings was positively associated with PTSD symptoms, *b* = 0.15, 95% CI = 0.07, 0.25, but was not associated with depression symptoms, *b* = 0.10, 95% CI = −0.01, 0.23. General experiences of discrimination were negatively associated with depression, *b* = −0.32, 95% CI = −0.38, −0.26, but were not associated with PTSD symptoms, *b* = −0.03, 95% CI = −0.08, 0.02. No other variable was associated with depression (i.e., all other confidence intervals included zero). Gender was associated with PTSD symptoms, such that women reported more symptoms than men, *b* = 1.23, 95% CI = 0.28, 2.23. No other variable was associated with PTSD symptoms (i.e., all other confidence intervals included zero). Additional statistical information can be found in Table 2.

### 5.2. Immigration Stress as a Predictor of PTSD and Depression

In the immigration stress model, immigration stress positively predicted PTSD symptoms, *b* = 0.12, 95% CI = 0.05, 0.18, but was not associated with depression symptoms, *b* = 0.004, 95% CI = −0.10, 0.10.

### 5.3. Direct Effects on Mental Health Access Variables

PTSD and Depression Symptoms as Predictors

In the discrimination model, PTSD symptoms were positively associated with having unsuccessfully sought mental health services, aOR = 1.18, *b* = 0.16, 95% CI = 0.03, 0.38, but depression symptoms were not significantly associated with having unsuccessfully sought services, *b* = −0.03, 95% CI = −0.18, 0.09. PTSD symptoms were also positively associated with currently receiving care, aOR = 1.16, *b* = 0.15, 95% CI = 0.01, 0.34. Depression symptoms were not significantly associated with currently receiving mental health services, *b* = 0.08, 95% CI = −0.06, 0.23. These results did not significantly differ in the immigration stress model.

### 5.4. Discrimination Variables as Predictors of Access Variables

General discrimination was not associated with either unsuccessfully seeking or currently receiving mental health services (confidence intervals include zero). Discrimination in healthcare settings was positively associated with currently receiving mental health services, aOR = 1.11, *b* = 0.10, 95% CI = 0.04, 0.21. Discrimination in healthcare settings was not associated with unsuccessfully seeking mental health services, aOR = 1.09, *b* = 0.09, 95% CI = −0.004, 0.19.

### 5.5. Immigration Stress as a Predictor of Access Variables

Immigration stress did not predict either currently receiving, aOR = 0.99, *b* = −0.01, 95% CI = −0.08, 0.06, or unsuccessfully seeking mental health services, aOR = 1.03, *b* = 0.03, 95% CI = −0.03, 0.10.

### 5.6. Indirect Effects with Symptomology as Mediators

Because the direct effects of discrimination on PTSD and PTSD on both mental health service access variables were significant, their resulting indirect effects were tested. A similar pattern was also observed for immigration stress with resulting indirect effects. The indirect effect of discrimination on unsuccessfully seeking care with PTSD as a mediator was significant and positive, *b* = 0.02, 95% CI = 0.01, 0.07. The indirect effect of discrimination on currently receiving care with PTSD as a mediator was also significant and positive, *b* = 0.02, 95% CI = 0.001, 0.05. The indirect effect of immigration stress on unsuccessfully seeking care with PTSD as a mediator was also significant, *b* = 0.11, 95% CI = 0.04, 0.19. The indirect effect on currently receiving care was also significant, *b* = 0.09, 95% CI = 0.02, 0.18.

## 6. Discussion

The current study examined the potential double-edged sword of discrimination and immigration-related stress. Specifically, the study examined the potential for two opposing effects of discrimination and immigration-related stress on care access: a direct pathway with a lower likelihood of seeking care and an indirect pathway with symptoms as a mediator and a greater likelihood of seeking care. Results testing these hypotheses were mixed. Specifically, general discrimination was only associated with depression symptoms but not PTSD. Conversely, healthcare discrimination and immigration stress were only associated with PTSD, but not depression. Further, discrimination in healthcare settings was associated with currently receiving care, but in the opposite direction than was anticipated. In fact, experiencing discrimination in healthcare settings was associated with *higher* odds of currently receiving care. General discrimination and immigration-related stress were not directly associated with either help-seeking variable.

These results provide novel data supporting only the indirect pathway of discrimination and immigration stress being associated with seeking or receiving mental health services. That is, both immigration-related stress and discrimination were associated with a *greater* likelihood of both attempting to receive care and successfully obtaining it. Further, these effects only occurred via PTSD symptoms and not depression. This fits with other research indicating that experiences of discrimination may uniquely predict PTSD symptoms [9]. However, it differs from prior work in that only discrimination in healthcare settings, but not more broadly, predicted PTSD. It is unclear why general discrimination was not associated with PTSD. It is also unclear why immigration stress was only associated with PTSD. It may be that immigration stress results in significant anxiety and avoidance, specifically. 

Other novel findings include that only PTSD and not depression predicted care seeking and access among this Latinx sample. The fact that only PTSD consistently predicted care seeking or access may be explained in multiple ways. First, PTSD may not be treated as successfully as depression, as prior work indicates that those with PTSD often do not receive adequate care, and Latinx populations often experience greater disparities [38]. Thus, depression and PTSD may both prompt service access, but depression may be successfully treated and thus not associated with receiving care. Alternatively, these results may reflect the difficulty in treating PTSD and/or the lack of access to appropriate care for PTSD. This, however, does not explain results in which depression did not predict unsuccessfully seeking care as unsuccessfully seeking care would not be presumed to result in symptom improvement, and depression symptoms would be expected to still correlate with attempts to seek care. Better explaining this pattern of results and linking to the Health Belief Model, depression may result in both greater perceived symptom severity and lower perceived effectiveness of treatment. In other words, they may recognize the symptoms as problematic, which should prompt greater help seeking, but not have confidence that treatment will improve these symptoms, which would prompt less help seeking according to the Health Belief Model. These novel insights regarding the different help-seeking effects of depression and PTSD offer fruitful avenues for future inquiry based on the Health Belief Model.

In addition to novel findings with the indirect effects of discrimination and immigration-related stress via PTSD, the current study did not support the direct effects of discrimination and immigration-related stress on care seeking. In fact, discrimination in healthcare settings was associated with a higher likelihood of having sought care. This directly conflicts with the Health Belief Model, especially given that discrimination in healthcare was associated with greater care access and seeking. One potential reason for the positive effect of healthcare discrimination on currently receiving care may be related to the cross-sectional nature of this study. Specifically, contact with the mental healthcare system or other referring healthcare systems may produce experiences of discrimination. Thus, those who access care may also be more likely to experience discrimination, which may, in turn, lead to worse PTSD symptomology. Longitudinal research is needed to explicate these relations fully. However, the pattern from the current cross-sectional data may reflect an underlying bidirectional relation in which PTSD increases the likelihood of seeking or receiving care, and contact with the healthcare system results in discrimination, which then worsens PTSD symptomology, leading to an increased need for care. Instead of a double-edged sword, healthcare discrimination may place some in a double bind where the system intended to help alleviate symptoms may worsen these symptoms when discrimination is present. These findings offer potential insights for future areas of inquiry, especially as they relate to the longitudinal roles of discrimination and immigration-related stress on care seeking. It further raises questions regarding the timing of such effects.

## 7. Limitations and Future Directions

The current study should be considered in the context of multiple limitations. First, the lack of longitudinal data makes it difficult to disentangle the effects of discrimination on healthcare access and to test mediation fully. Second, the study’s convenience sample may limit generalization to other Latinx populations. While the inclusion of a sample recruited outside of any major metropolitan areas may extend the literature, healthcare systems may differ significantly in these areas, including the extent to which people experience discrimination and other available resources. Relatedly, the sample includes primarily cisgender women, and the sample size was insufficient to test gender differences across the effects examined here. Given gender differences in both symptomology and help seeking, results may differ for cisgender men and trans or gender-diverse populations. However, the current results point to potentially fruitful areas of investigation, including longitudinal examination of discrimination, healthcare access, and help-seeking behavior. Future studies should examine different timescales over which discrimination and immigration-related stress predict help seeking. For example, examining whether experiencing discrimination in healthcare settings results in reduced help-seeking attitudes or behaviors over shorter (e.g., days) and longer (e.g., months or days) time intervals. The current study, combined with prior data, indicates that such investigations may shed important light and nuance onto the role of discrimination for Latinx populations in the U.S. Additionally, these results should be examined in different geographical contexts, as they may differ in contexts depending on how welcoming the community is to immigrants and the availability of services.

## 8. Conclusions

The current study suggests that discrimination in healthcare settings and immigration-related stress may increase PTSD symptoms, which in turn may prompt attempts to access care. The same effects were not present for depression symptoms. This offers new insights into the unique role of PTSD symptoms as consequences of stressors among Latinx populations and immigrants in particular. This novel and somewhat counterintuitive contribution indicates that both discrimination and immigration-related stress may indirectly increase service seeking because both may increase PTSD symptoms.

## Figures and Tables

**Table 1:** Demographic and descriptive information.

	*n* (%)	
Gender		
Cisgender women	165 (70.2)	
Cisgender men	65 (27.7)	
Transgender men	1 (0.4)	
Born outside the U.S.	197 (83.8)	
Mexico	91 (38.7)	
Caribbean	39 (16.6)	
Central America	45 (19.1)	
South America	23 (9.8)	
Education		
Less than high school	50 (21.3)	
Completed high school	86 (36.6)	
Some college or higher	92 (39.1)	
Unsuccessfully sought mental health care	21 (8.9)	
Currently receiving care (yes)	21 (8.9)	
	M (SD)	Min-Max
Age	42.52 (14.21)	19–82
Annual household income (in USD)	42,596 (33,963)	0–200,000
PHQ-8 (depression symptoms)	7.12 (5.50)	0–24
Abbreviated PCL-5 (PTSD symptoms)	2.41 (3.11)	0–14
Everyday discrimination	5.26 (4.88)	0–20
Discrimination in healthcare	12.09 (2.98)	4–20
Immigration-related stress	38.15 (9.21)	16–58

**Table 2:** Predictors of mental health access variables.

Predictor	Dependent Variables	
	Unsuccessfully Seeking Care	Current Care Utilization
	aOR	aOR
Gender	0.85	0.77
Age	0.78	1.10
Everyday discrimination	1.00	1.01
Healthcare discrimination	1.11 *	1.09 *
Depression symptoms (PHQ-8)	0.97	1.08
PTSD symptoms (PCL)	1.16 *	1.18 *
Immigration-related stress	1.03	0.99

Note: * Significance determined using bootstrapped confidence intervals of regression coefficients.

## Data Availability

Data are not available for sharing due to privacy concerns of participants. Additional data summaries may be provided upon request.
